# *Chrysanthemum morifolium* and Its Bioactive Substance Enhanced the Sleep Quality in Rodent Models via Cl^−^ Channel Activation

**DOI:** 10.3390/nu15061309

**Published:** 2023-03-07

**Authors:** Mijin Kim, YuJaung Kim, Hyang Woon Lee, Jae-Chul Jung, Seikwan Oh

**Affiliations:** 1 Department of Molecular Medicine, School of Medicine, Ewha Womans University, Seoul 07804, Republic of Korea; 2 Department of Neurology, Medical Research Institute, School of Medicine, Ewha Womans University, Seoul 07804, Republic of Korea; 3 Graduate Programs in Artificial Intelligence Convergence, Computational Medicine, System Health Science and Engineering, Ewha Womans University, Seoul 03765, Republic of Korea; 4 Life Science Research Institute, NOVAREX Co., Ltd., Cheongju 28220, Republic of Korea

**Keywords:** sleep, linarin, pentobarbital, electroencephalogram, GABA_A_ receptor

## Abstract

Dried *Chrysanthemum morifolium* (Chry) flowers have been used in Korea as a traditional insomnia treatment. In this study, the sleep-promoting activity and improving sleep quality of Chry extract (ext) and its active substance linarin were analyzed by pentobarbital-induced sleep experiment in mice and electroencephalography (EEG), electromyogram (EMG) analysis in rats. In a dose-dependent manner, Chry ext and linarin promoted longer sleep duration in the pentobarbital-induced sleep test compared to pentobarbital-only groups at both hypnotic and subhypnotic doses. Chry ext administration also significantly improved sleep quality, as seen in the relative power of low-frequency (delta) waves when compared with the control group. Linarin increased Cl^−^ uptake in the SH-SY5Y human cell line and chloride influx was reduced by bicuculline. After administration of Chry ext, the hippocampus, frontal cortex, and hypothalamus from rodents were collected and blotted for glutamic acid decarboxylase (GAD)_65/67_ and gamma-aminobutyric acid (GABA)_A_ receptors subunit expression levels. The expression of α1-subunits, β2-subunits, and GAD_65/67_ of the GABA_A_ receptor was modulated in the rodent brain. In conclusion, Chry ext augments pentobarbital-induced sleep duration and enhances sleep quality in EEG waves. These effects might be due to the activation of the Cl^−^ channel.

## 1. Introduction

Humans spend about a third of their lives sleeping, but relatively little is known about why this process is so important to all living creatures. Insufficient sleep can be a determinant of the development of serious medical conditions and is associated with a higher risk of death [1]. Sleep is known to be important in regulating physiological processes in the body [2,3]. Deficiency of sleep causes not only physical problems such as diabetes, atherosclerosis, and neurodegeneration [4] but also mental health problems, especially anxiety and depression [5]. Therefore, sufficient sleep is essential, and interest in individual lifestyles for controlling optimal sleep quality within sleeping hours and overcoming sleep disorders is increasing.

Drugs developed to treat sleep disorders, such as benzodiazepine drugs, imidazopyridine drugs, and barbiturates, induce and maintain sleep by suppressing the central nervous system (CNS) [6,7,8,9]. It is known that the representative mechanism of inhibition is central to the regulation of gamma-aminobutyric acid (GABA) transmission and involves binding to GABA_A_ receptor subtypes. GABA_A_ receptor agonists that enhance Cl^−^ uptake also augment Cl^−^ uptake when administered with pentobarbital or other agonists [10,11]. However, the drugs may have undesirable side effects [12]. Therefore, interest in sleep aids without unwanted side effects is needed.

*Chrysanthemum morifolium* (Chry), mainly available in Asian countries, has been widely used as a medicinal herbal tea in oriental countries for hundreds of years [13]. The medicinal effects of Chry affect the central nervous system, and it is used as an insomnia treatment [14], anticonvulsant, and sedative [15]. It is also reported to have various medicinal properties, such as antioxidant [16,17], anti-arthritic [18], and anti-inflammatory [19] properties. The medicinal effect of Chry is attributed to its significant flavonoids as its main active ingredients, among which linarin is the main flavone [20,21].

According to the reports, Chry extract (ext) showed the effect of increasing sleep time in mice [14]. However, the exact components of Chry and the underlying neurological mechanisms responsible for Chry’s effect in increasing sleep duration remain unknown. In addition, there is little data on the effect of EEG wavelength on the sleep stage in rats. Therefore, this study aimed to prove that Chry ext can not only increase sleep duration but also improve sleep quality and to identify the active mechanism of Chry for improving sleep quality. In this study, the improvement of sleep quality of the active ingredient and extract was evaluated.

## 2. Materials and Methods

### 2.1. Chemicals

*Chrysanthemum morifolium* 50% ethanol extract (Chry ext) was obtained from NOVAREX Co., Ltd. (Cheongju, Republic of Korea). Pentobarbital sodium was obtained from Hanlim Pharm (Seoul, Republic of Korea). Antibodies against GABA_A_ receptor α1 (NB300-191), β2 (NB300-198), and γ2 (NB300-151) were purchased from Novus biologicals (Littleton, CO, USA). Antibody against GAD_65/67_ (sc-365180) was obtained from Santa Cruz Biotechnology (Dallas, TX, USA). HRP-linked anti-rabbit IgG (#7074) and anti-mouse IgG (#7076) secondary antibodies were purchased from Cell Signaling Technology (Danvers, MA, USA). N-(Ethoxycarbonylmethyl)-6-Methoxyquinolinium Bromide (MQAE) was purchased from Thermo Fisher Scientific Inc. (Waltham, MA, USA). Other chemicals were purchased from Sigma-Aldrich (St. Louis, MO, USA).

### 2.2. Animals

Male ICR mice (32–35 g) and Sprague-Dawley (SD) rats (male, 280–300 g) were purchased from RaonBio (Yongin, Republic of Korea). Animals were group-housed (mice, 5/cage; rats, 2/cage) and maintained in an ambient atmosphere at 21–23 °C under a 12:12 h light–dark cycle allowing free access to water and food. All the experiments were performed in accordance with institutional guidelines, and the protocol was approved by the Institutional Animal Care and Use Committee of Ewha Womans University School of Medicine (EWHA MEDIACUC past-040-4, 22-025).

### 2.3. Pentobarbital-Induced Sleeping Test

Sleep was evaluated based on the prolongation of the pentobarbital-induced sleeping time by the treatments. Sleep evaluation was performed between 10:00 and 16:00 as previously described [22,23,24] with slight modification. Two separate different doses of pentobarbital were used. The first dose was a subhypnotic dose of 28 mg/kg of pentobarbital, and the second dose was a hypnotic dose of 42 mg/kg of pentobarbital. For Chry treatment, mice were divided into 4 groups for each pentobarbital dose (pentobarbital only, pentobarbital + Chry 50 mg/kg, pentobarbital + Chry 100 mg/kg, pentobarbital + Chry 200 mg/kg, n = 10). For linarin treatment, mice were divided into 3 groups for each pentobarbital dose (pentobarbital only, pentobarbital + linarin 5 mg/kg, pentobarbital + linarin 10 mg/kg, n = 10).

Briefly, the mice were administered the saline (control), Chry ext (50, 100, and 200 mg/kg, p.o.), or linarin (5 and 10 mg/kg, i.p.) twice 24 h apart. One hour after the last treatment, sleep was induced by injecting pentobarbital. The mice were in a state of sleep if the mice stayed motionless and lost their righting reflex when oriented on their back (up to a maximum of 2 h). Sleep onset time was recorded as the time interval between pentobarbital injection and falling asleep. Mice were excluded if they did not sleep within 15 min after the pentobarbital injection.

### 2.4. Electroencephalography (EEG) and Electromyogram (EMG) Implantation Surgery

**Figure 1:**
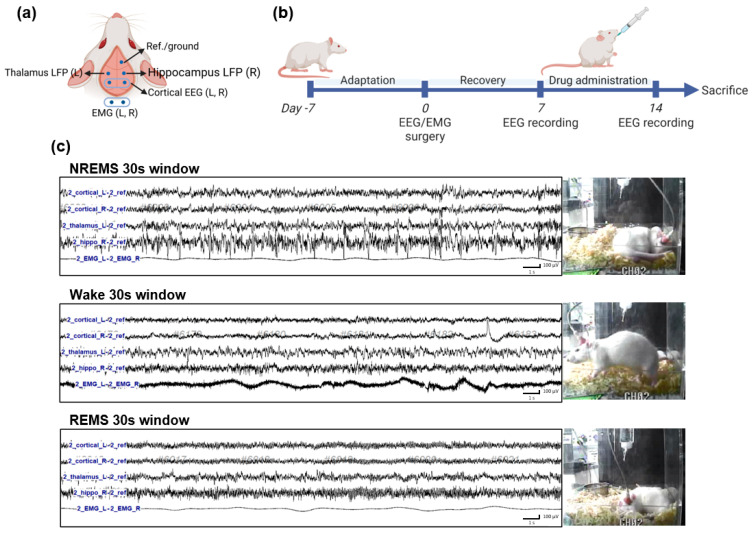
Schematic diagram of electrode placement for EEG/EMG recording and reference in the rat skull (**a**) and experimental design and timelines for the EEG/EMG recordings (**b**). Typical EEG/EMG waveforms are displayed for 30 s in each sleep state in rats (**c**). EEG, electroencephalogram; EMG, electromyogram; LFP, local field potential; REMS, rapid eye movement sleep; NREMS, non-REMS; Wake, wakefulness.

All surgeries were performed in a dedicated stereotaxic frame under inhaled isoflurane anesthesia (2–5% in O_2_). EEG and EMG electrodes were implanted in rats (n = 6) as described previously [25,26,27,28]. A schematic diagram of the EEG/EMG coordinates is shown in Figure 1a. Briefly, a reference ground screw was placed on the frontal area, and two EEG screws were placed on the bilateral cerebral cortex. Two depth electrodes were placed in the left thalamus (from bregma: AP = −3.0 mm, ML = −3.0 mm, DV = −6.4 mm) and in the right hippocampus (from bregma: AP = −2.8 mm, ML = +1.8 mm, DV = −3.3 mm). The two EMG electrodes were constructed from 15 mm of nylon-insulated silver wire (0.013” coated, A-M Systems, Sequim, WA, USA) and were sutured into the superior nuchal muscles in the back of the neck (Figure 1a). All seven EEG/EMG leads were soldered to the female contacts (E363/0; P1 technologies) and were inserted into a 7-channel pedestal (MS7Pl; P1 technologies). The base of the pedestal and screw heads were secured with dental acrylic (self-curing set; Vertex Dental BV, Zeist, Netherlands), and the skin was sutured, leaving only the pedestal socket exposed. Rats received post-operative analgesia (ketoprofen, 2.5 mg/kg, i.p.) and were housed individually in cages to recover for 1 week before recordings were made.

### 2.5. EEG Recording and Sleep–Wake State Analysis

For EEG recording, the rats were divided into 3 groups (control, Chry 100 mg/kg, Chry 200 mg/kg, n = 6). To record EEG and EMG, a 7-pin EEG/EMG cable (363–363; P1 Technologies) was inserted into the implanted pedestal, then connected to a commutator. To allow the rats to get accustomed to the tethered environment, each animal was placed in the experimental apparatus for 3 days prior to the recording. EEG/EMG signals were continuously collected and saved at 200 Hz sampling rates through a recording amplifier (AURA24; Grass-Telefactor, West Warwick, RI, USA) and data acquisition software (Twin 4.5.3; Grass Technologies LTM). Sleep state recordings were made twice, one day before saline (control) or Chry ext treatment (100, and 200 mg/kg) and one hour after the last treatment for 7 days, as shown in the timeline in Figure 1b. Considering the circadian pattern of rodents, 6 h (10:00 to 16:00) were exported and used for analysis. The sleep–wake states (wakefulness, REM, and NREM sleep) were scored semi-automatically by 5 s epochs of EEG/EMG signals using the open-source software AccuSleep [29] written in MATLAB (R2021b, The MathWorks Inc., Natick, MA, USA). All results of the AccuSleep scoring were validated after visual analysis to reduce errors. Representative EEG/EMG waveforms are shown in Figure 1c.

### 2.6. Time-Dependent Power Spectrum Heatmaps

Time–frequency power spectrum density (PSD) that reflects the power of the EEG signals was calculated based on a short-time Fourier transform between 0–20 Hz with a 5 s window and 0.25 s step. The PSD values and the percentage power density were displayed in three different frequency bands, namely delta (0.5–4 Hz), theta (4–8 Hz), and alpha (8–13 Hz).

### 2.7. Western Blotting

The selected brain samples (frontal cortex, hippocampus, and hypothalamus) were collected after the behavior test and EEG recording. Samples were lysed with RIPA buffer (ELPIS Biotech Inc., Daejeon, Republic of Korea). The proteins were separated by 12% SDS-PAGE electrophoresis, transferred to a PVDF membrane, blocked with 5% skim milk, and were incubated at 4 °C with the primary antibodies (1:1000) overnight and with the secondary antibodies (1:2000) for 1 h. The proteins were detected using enhanced chemiluminescence (ECL) detection kits (Bio-Rad, Hercules, CA, USA).

### 2.8. Intracellular Chloride Ion Measurement Assay

Based on previous studies [22,30], relative changes were measured using N-(Ethoxycarbonylmethyl)-6-Methoxyquinolinium Bromide (MQAE), a Cl^−^-sensitive indicator, in SH-SY5Y human neuroblastoma cell line obtained from the Korea cell line bank (Seoul, Republic of Korea). Cells (4 × 10^5^ cells/mL) were incubated with 5 mM MQAE in Cl^−^ free buffer in a 96-well plate for 3 h. Then, the cells were washed five times and treated with 500 µM bicuculline or 12.5 µM flumazenil for 15 min at room temperature. The plate was read at extinction of 365 nm and emission of 450 nm using a fluorescent reader (Synergy H1, BioTek Instruments Inc., Winooski, VT, USA). Data are presented as relative fluorescence F/F_0_, where F is the fluorescence as a function of time, and F_0_ is the fluorescence without Cl^−^ ions. The F/F_0_ values are directly proportional to [Cl^−^]_i_.

### 2.9. Statistical Analysis

Statistical analysis was performed using Graph Prism 5 software (GraphPad Software Inc., San Diego, CA, USA). Values were expressed as means ± standard error of the mean (SEM), and statistical significance was accepted for *p* values < 0.05. Data were analyzed using one-way analysis of variance (ANOVA) with Tukey’s post hoc test and two-way ANOVA with Bonferroni post hoc test.

## 3. Results

### 3.1. Extract of Chrysanthemum morifolium and Its Bioactive Substance Enhances Sleep Duration in Pentobarbital-Induced Sleep in Mice

**Figure 2:**
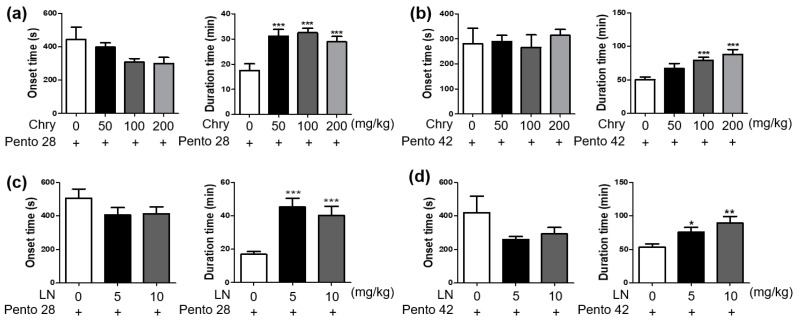
Effects of Chry and linarin on sleep onset and sleep duration in pentobarbital-treated mice. The experiment design; mice were fasted for 24 h before the experiment, and Chry ext or linarin was administered at each concentration 1 h and 25 h before the intraperitoneal administration of pentobarbital. Sleep onset and sleep duration were assessed. The results of Chry ext tested at pentobarbital subhypnotic (**a**) and hypnotic (**b**) concentrations are displayed. The results of linarin tested at pentobarbital subhypnotic (**c**) and hypnotic (**d**) concentrations are displayed. Data are expressed as mean ± SEM. * *p* < 0.05, ** *p* < 0.01, and *** *p* < 0.001, compared with the pentobarbital only group. Chry, *Chrysanthemum morifolium* extract; Pento, pentobarbital; LN, linarin.

Pentobarbital induced sleep in a dose-dependent manner. When only pentobarbital was given, sleep duration was about 17 min at 28 mg/kg pentobarbital dose and about 52 min at 42 mg/kg pentobarbital dose. In sleep experiments using the subhypnotic concentration of 28 mg/kg pentobarbital, mice pretreated with *Chrysanthemum morifolium* extract (Chry ext) showed no significant change in onset time in all groups but had prolonged sleep duration by up to 41 min when compared to the pentobarbital-only group (Figure 2a). Similar results were observed in mice that received the hypnotic concentration of 42 mg/kg pentobarbital and pretreated with Chry ext. Pretreatment with Chry ext increased sleep duration dose-dependently in mice given 42 mg/kg pentobarbital (Figure 2b). With both pentobarbital doses, Chry ext administration increased sleep duration without significant change in sleep onset time.

Linarin is one of the bioactive substances of Chry. In sleep experiments using 28 mg/kg and 42 mg/kg pentobarbital, linarin also increased sleep duration in mice (Figure 2c,d), consistent with the previous sleep experiments using Chry ext pretreatment (Figure 2a,b). Sleep onset was also shortened by linarin in both doses of pentobarbital without statistical significance.

### 3.2. Extract of Chrysanthemum morifolium Improves Sleep Quality in Rats

**Figure 3:**
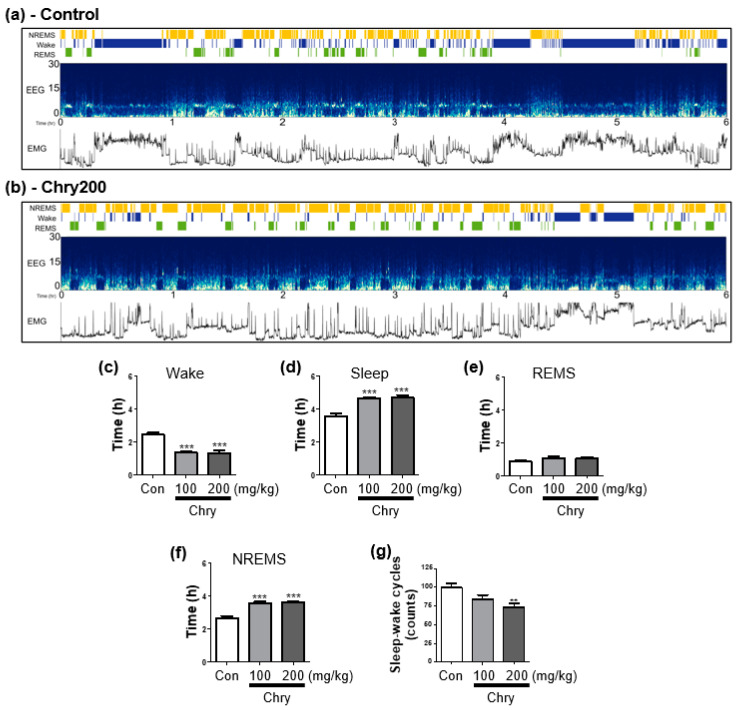
Sleep analysis of rats through automated sleep scoring by AccuSleep algorithm in MATLAB. After the administration of Chry ext (100 and 200 mg/kg, p.o.) once a day for 7 days, EEG and EMG recorded for 6 h (10:00–16:00) at the last administration were used. The representative examples of AccuSleep interface including the sleep stages, EEG spectrogram, and EMG power in control and Chry200 are displayed (**a**,**b**). Wake and sleep time analysis (**c**,**d**), duration of REMS and NREMS time (**e**,**f**), and sleep–wake cycles (**g**) were determined. Data are expressed as mean ± SEM. ** *p* < 0.01, and *** *p* < 0.001, compared with the control group. Chry, *Chrysanthemum morifolium* extract; EEG, electroencephalogram; EMG, electromyogram; REMS, rapid eye movement sleep; NREMS, non-REMS; Wake, wakefulness.

EEG/EMG recordings obtained from electrode-implanted rats were used for sleep analysis. A representative overall flow of EEG/EMG recordings for 6 h (10:00–16:00) is shown in Figure 3a,b through AccuSleep’s interface using MATLAB. Chry ext (100, and 200 mg/kg) administration groups had decreased awake time and increased sleep duration in the recorded 6 h when compared to the control group (Figure 3c,d). The sleep time was subdivided into rapid eye movement sleep (REMS) and non-REMS (NREMS) through EEG analysis. There was no significant effect on the temporal distribution of REMS (Figure 3e), but the time of NREMS was highly increased (Figure 3f). High-dose treatment of Chry ext (200 mg/kg) also significantly decreased sleep–wake cycles (Figure 3g).

### 3.3. Extract of Chrysanthemum morifolium Improves Sleep Quality in Power Spectrogram Analysis

**Figure 4:**
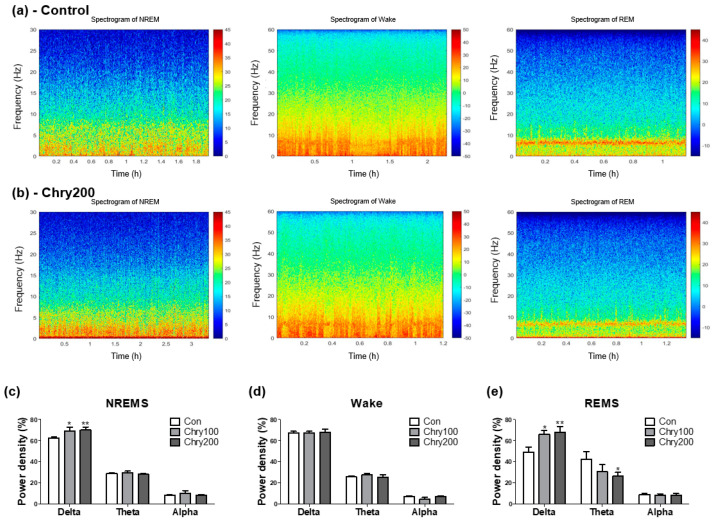
Time-dependent power spectrum heatmaps. After the administration of Chry ext (100 and 200 mg/kg) once a day for 7 days, EEG/EMG recorded for 6 h (10:00–16:00) at the last administration were used. The representative power spectrum heatmaps, drawn with time on the *X*-axis and frequency on the *y*-axis, show the representative figures showing the characteristics of each group’s REMS, Wake, and NREMS states (**a**,**b**). Jet colormap was used for the data range displayed on the right side of the heatmap, and the range of expression was adjusted so that the characteristics of each state were clearly displayed. % of EEG power spectrum densities during NREMS (**c**), Wake (**d**), and REMS (**e**). Data are expressed as mean ± SEM. * *p* < 0.05, and ** *p* < 0.01, compared with the control group. Chry, *Chrysanthemum morifolium* extract; REMS, rapid eye movement sleep; NREMS, non-REMS; Wake, wakefulness.

Figure 4a,b show representative time-dependent power spectrum heatmaps for each group in wake, REMS, and NREMS states. Figure 4c–e show the calculated average of the power densities corresponding to the delta (δ; 0.5–4 Hz), theta (θ; 4–8 Hz), and alpha (α; 8–13 Hz) wavelength bands as a percentage of these three wavelengths. The percentage of delta power density was significantly enhanced by treatment with Chry ext (100 and 200 mg/kg) during NREM and REM sleep. The theta frequency band showed a negligible difference between NREMS and wake, whereas in REMS, it showed a slight weakening trend when treated with a high concentration of Chry ext. There was no change in all three wavelengths during wake time.

### 3.4. Extract of Chrysanthemum morifolium Modulates Expression of Subtypes of GABA_A_ Receptors in the Rat Brain

**Figure 5:**
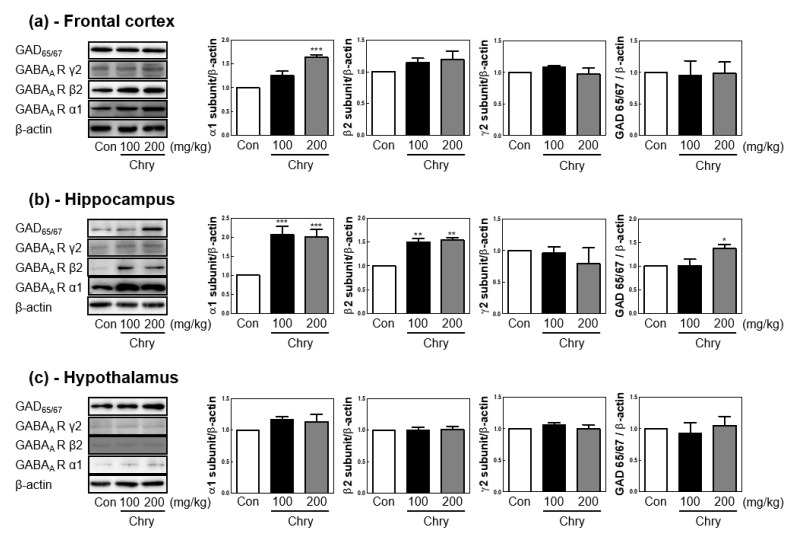
Extract of Chry modulates glutamic acid decarboxylate (GAD)_65/67_ and gamma-aminobutyric acid (GABA)_A_ receptor subunits. Western blot analysis of (**a**) rat frontal cortex, (**b**) hippocampus, and (**c**) hypothalamus after EEG analysis. The representative results from three independent experiments are shown, and quantification data are at the right panel. Data are expressed as mean ± SEM. * *p* < 0.05, ** *p* < 0.01, and *** *p* < 0.001, compared with the control group.

GABA_A_ receptor subtypes in neural tissue are reported to be targets for the treatment of insomnia [31,32]. After the EEG recording, the rats were sacrificed, and the brain was extracted immediately. We isolated the frontal cortex, hippocampus, and hypothalamus from the brain and investigated whether protein expression of GABA_A_ receptor subunits α1, β2, and γ2 was regulated in each region. In the frontal cortex, treatment with 200 mg/kg of Chry ext increased α1 protein expression (Figure 5a), and in the hippocampus, both 100 and 200 mg/kg of Chry ext increased the protein expression of α1 and β2 (Figure 5b). It also increased glutamic acid decarboxylase (GAD)_65/67_ protein expression levels. The hypothalamus showed no change in the different GABA_A_ receptor subtypes protein expression levels (Figure 5c).

### 3.5. Linarin Activates the Chloride Ion Channels of GABA_A_ Receptors

**Figure 6:**
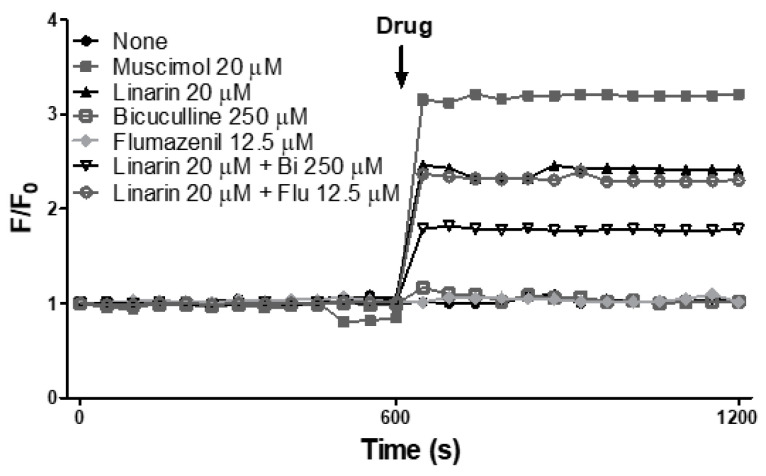
Linarin shows Cl^−^ influx in human neuroblastoma cells (SH-SY5Y) in vitro. Cells were incubated with 5 mM N-(Ethoxycarbonylmethyl)-6-Methoxyquinolinium Bromide (MQAE) for 3 h and then treated with 500 µM bicuculline or 12.5 µM flumazenil for 15 min. Fluorescence was monitored using excitation at 365 nm and emission at 450 nm. Contents of influxed Cl^−^ ion were expressed as a peak (F/F_0_).

Figure 6 shows the effect of linarin on intracellular Cl^−^ influx using the human SH-SY5Y neuroblastoma cell line in vitro. The concentration used in the experiment was referred from previous reports and determined using cell viability assay and cell cytotoxicity assay. Bicuculline is a competitive GABA_A_ receptor antagonist, and flumazenil is a benzodiazepine receptor antagonist. Muscimol was used as a positive control as a potent GABA_A_ receptor agonist. Pretreatment with antagonists alone did not significantly change MQAE fluorescence values. Treatment with linarin alone caused a decrease in MQAE fluorescence value similar to muscimol treatment, indicating Cl^−^ influx was modulated. Pretreatment with flumazenil before linarin treatment did not significantly affect intracellular Cl^−^ influx compared to linarin alone, but pretreatment with bicuculline inhibited the influx of intracellular Cl^−^ by linarin. These results showed that linarin elevated the Cl^−^ influx via GABA_A_ receptor activation.

## 4. Discussion

Although the etiology of sleep disorders is not fully understood, characteristic features of insomnia are difficulties in falling asleep, maintaining sleep, and waking up from sleep. Several drugs have been developed to improve insomnia, but many of these still cause serious side effects. For that reason, the development of alternative supplements from natural products with fewer side effects is on the rise. *Chrysanthemum morifolium* (Chry) has been traditionally used in the Orient as a tea-type intake method for helping with sleep. It contains various active flavonoids, including flavone substances such as linarin [15]. Chry has been reported to have a soothing effect, but there are not many explanations about the mechanism and identification of the main soothing component of Chry, especially the part related to electroencephalogram (EEG) and electromyogram (EMG). The purpose of this paper was to find out whether the extended sleep duration effect of Chry extract (ext) is related to sleep quality improvement and to explain its sleep quality improvement effect from a neurological mechanism viewpoint.

The pentobarbital-induced sleep mouse model is the most commonly used method for screening hypnotics [33,34]. As a result of the pentobarbital-induced sleep behavior experiment, Chry ext and its bioactive substance, linarin, showed a pattern of reducing sleep onset without statistical significance. However, Chry ext and linarin enhanced the pentobarbital-induced sleep behavior of mice. Linarin elevating sleep duration suggests that linarin plays an important role in the sleep-prolonging effect of Chry. When pentobarbital was not administered, the sleep duration in rats was not increased by Chry ext or linarin alone (data are not shown), demonstrating that Chry ext and linarin by themselves were unable to induce sleep even at high doses, unlike sleeping pills. This suggests that it works as a sleep aid and that Chry ext or linarin has an important effect on enhancing the induced hypnotic effect. Pentobarbital activates the gamma-aminobutyric acid (GABA)_A_ receptor in the central nervous system (CNS), induces the opening of chloride channels, and strengthens the GABA effect to produce sedative and hypnotic effects [35]. Many hypnotic, anxiolytic, and antiepileptic drugs prolong the sleep duration induced by pentobarbital [36,37]. Thus, it suggests that Chry ext can be a helpful agent in prolonging sleep duration as a result of its CNS depressant effect in rodents and supports earlier observations that Chry plants are sedatives.

Sleep is divided into two broad stages: rapid eye movement sleep (REMS) and non-REMS (NREMS) [38]. Stages of sleep can be monitored and measured by EEG/EMG analysis. EEG is an electrophysiological monitoring method that records electrical activity by measuring voltage fluctuations caused by ionic currents within neurons in the brain [39]. EEG parameters are used to assess sleep patterns or sleep quality [40,41,42]. Through a pentobarbital-induced sleep experiment, the dose-response relationship of Chry ext was confirmed, and EEG/EMG analysis was performed after selecting an effective dose. In the representative AccuSleep interface of each group, it can be confirmed with the naked eye that the control group has a high wake rate. As a result of EEG analysis, administration of Chry, ext decreased the duration of wakefulness during the total 6 h recording compared to control and increased sleep duration supporting the pentobarbital-induced sleep test results. In addition, there was no significant change in the REMS time but a significant increase in NREMS time. When assessing good sleep, it is important to evaluate the quality of sleep as well as the amount of sleep. Administration of Chry ext reduced the frequency of awakenings during sleep. Although sleep quality is difficult to define in rodents, an important indicator of sleep quality is the depth or intensity of NREMS using the delta activity observed in the deep phase of NREMS [43]. As another method to demonstrate the improvement in sleep quality, power density for each frequency band was analyzed. Unlike the well-known hypnotics, zolpidem, which showed reduced delta activity [44], Chry ext showed a tendency to increase delta activity during NREMS without affecting theta activity. In addition, there was no change in power density for the wake state. The ideal sleep aid is one that does not affect wakefulness after sleep, and Chry ext appears to have this function. As a result, several analyses of EEG/EMG recordings demonstrated that Chry ext not only increased sleep time but also improved sleep quality.

Various neurotransmitters, including GABA, serotonin, adenosine, and histamine, are involved in regulating the switch between wakefulness and sleep [45,46,47]. Since pentobarbital-induced sleep is enhanced through the GABAergic system [48,49], we were interested in confirming whether the sleep-prolonging effect of Chry ext pretreatment was due to its effect on the CNS through the GABAergic system. The hippocampus and frontal cortex are one of the main nodes that control memory recovery during sleep and behaviors related to anxiety, and the hypothalamus is an arousal system considered to play a role in regulating wakefulness. Among the several parts of the brain related to sleep, the hippocampus, frontal cortex, and hypothalamus were selected to measure protein expression levels [10,50,51,52,53]. The protein expression of the GABA_A_ receptor subunit and GAD_65/67_, which catalyzes the formation of GABA in neural tissue, were analyzed in the frontal cortex, hippocampus, and hypothalamus after 7 days of Chry ext administration. Chry ext induced modulation of the alpha1 subunit in the frontal cortex and alpha1, beta2, and GAD_65/67_ in the hippocampus. Further studies are needed to determine the elevated significance of each GABA_A_ receptor subunit. However, according to reports [54,55], GABA_A_ receptors are assembled from large family subunit genes by forming heteromeric GABA-gated Cl^−^ channels and have several binding sites, including GABA, benzodiazepine, and barbiturate sites. GABA_A_ receptor channels open after ligand binding, providing a net inward flux of negative Cl^−^ ions, which hyperpolarizes the membrane and reduces neuronal firing, thereby promoting sleep. Previous studies have shown that Cl^−^ uptake of GABA_A_ receptors is enhanced by drugs and other GABA_A_ receptor agonists [56]. In the human SH-SY5Y neuroblastoma cell line, linarin (a single substance of Chry ext) enhanced intracellular Cl^−^ influx, which was inhibited by bicuculline. Given that influx was not completely reversed by bicuculline, GABAergic modulation of Chry ext is likely to be allosteric modulation. Chry ext alone was unable to induce sleep, but it potentiated pentobarbital-induced sleep suggesting that Chry and pentobarbital have differences in their pharmacological properties for GABA_A_ receptors. In other words, Chry ext can modulate GABA_A_ receptors, induce Cl^−^ channel opening, and enhance hypnotic properties like a GABA_A_ receptor agonist.

To explain the sleep-enhancing effect of Chry ext with another approach, further research on spindle waves emitted from the thalamus is underway. Nevertheless, this study will help understand the role and mechanisms of Chry ext and its active substance, linarin, in improving sleep duration. While drugs used to treat sleep disorders have many side effects, natural product-derived Chry ext improves sleep quality as well as increasing sleep duration in rodents. Considering the importance of nutrition-based hypnosis in the treatment of sleep disorders, this study on Chry has advantages compared to other therapeutic drugs, suggesting the possibility of Chry ext as a dietary supplement for the treatment of sleep disorders. In addition, it can be a reference standard when discovering functional food as sleep aids.

## 5. Conclusions

The present data demonstrated that Chry ext can potentially improve sleep in normal rodents without affecting wake activity. Chry ext and linarin have potential hypnotic effects that are time- and dose-dependent and may be involved in the regulation of the GABAergic system through GABA receptors. In particular, Chry ext improves sleep quality as it increases the intensity of NREMS, and this effect was found to be due to the modulation of the GABAergic system. Given the global trend of sleep disorders, Chry ext as a sleep aid presents a new direction for developing new dietary supplements in the future.
